# The actomyosin interface contains an evolutionary conserved core and an ancillary interface involved in specificity

**DOI:** 10.1038/s41467-021-22093-4

**Published:** 2021-03-25

**Authors:** Julien Robert-Paganin, Xiao-Ping Xu, Mark F. Swift, Daniel Auguin, James P. Robblee, Hailong Lu, Patricia M. Fagnant, Elena B. Krementsova, Kathleen M. Trybus, Anne Houdusse, Niels Volkmann, Dorit Hanein

**Affiliations:** 1 Structural Motility, Institut Curie, CNRS, UMR 144, Paris, France; 2 Scintillon Institute, San Diego, CA USA; 3 Laboratoire de Biologie des Ligneux et des Grandes Cultures (LBLGC), Université d’Orléans, INRAE, USC1328, Orléans, France; 4 Department of Molecular Physiology & Biophysics, University of Vermont, Burlington, VT USA; 5 Structural Image Analysis Unit, Department of Structural Biology & Chemistry, Institut Pasteur, Paris, France; 6 Structural Studies of Macromolecular Machines in Cellulo Unit, Department of Structural Biology & Chemistry, Institut Pasteur, Paris, France

**Keywords:** Cryoelectron microscopy, Motor protein function

## Abstract

*Plasmodium falciparum*, the causative agent of malaria, moves by an atypical process called gliding motility. Actomyosin interactions are central to gliding motility. However, the details of these interactions remained elusive until now. Here, we report an atomic structure of the divergent *Plasmodium falciparum* actomyosin system determined by electron cryomicroscopy at the end of the powerstroke (Rigor state). The structure provides insights into the detailed interactions that are required for the parasite to produce the force and motion required for infectivity. Remarkably, the footprint of the myosin motor on filamentous actin is conserved with respect to higher eukaryotes, despite important variability in the *Plasmodium falciparum* myosin and actin elements that make up the interface. Comparison with other actomyosin complexes reveals a conserved core interface common to all actomyosin complexes, with an ancillary interface involved in defining the spatial positioning of the motor on actin filaments.

## Abstract (web summary)

*Plasmodium falciparum* moves by an atypical process called gliding motility which comprises of atypical myosin A (PfMyoA) and filaments of the dynamic and divergent PfActin-1 (PfAct1). Here authors present the cryo-EM structure of PfMyoA bound to filamentous PfAct1 stabilized with jasplakinolide and provide insights into the interactions that are required for the parasite to produce the force and motion required for infectivity.

## Introduction

Malaria is the deadliest parasitic disease around the world, responsible for a half-million deaths per year^1^. *Plasmodium falciparum* (Pf), the causative agent of malaria, is able to develop resistance to most of the treatments available today, creating the need for new therapeutic targets^2^. Like other Aplicomplexan parasites, *P. falciparum* moves by an atypical process called gliding motility^3^. The macromolecular complex necessary for force and motion production is called the glideosome, comprised of an atypical myosin A (PfMyoA) and filaments of the dynamic and divergent PfActin-1 (PfAct1)^4^. While the role of PfMyoA in the movement of the parasite had been established^5^, it was only recently shown that PfMyoA is essential for invasion of red blood cells by merozoites, the stage responsible for the symptoms of malaria^3^. PfMyoA thus represents an efficient pharmaceutical target since blocking its activity would impair both the motile and invasive stages of the parasite^6,7^.

PfMyoA is a divergent myosin, sharing only 28.66% identity with non-muscle myosin IIc (NM2c). Crystallographic structures of PfMyoA revealed an unforeseen N-term extension of the heavy chain that extends over the N-terminal subdomain surface and that can be phosphorylated on serine 19. Phosphorylation specifically tunes the properties of PfMyoA, which can work in two regimes: phosphorylated PfMyoA moves actin at high speed but has low ensemble force; unphosphorylated PfMyoA produces more force but at the expense of speed^6,8^. These results led us to consider PfMyoA as a tunable molecular engine that could function optimally either at high speed in highly mobile stages such as sporozoites that need to move at more than 2 µm s^−1^, or with high force in invasive stages such as merozoites that produce forces of ~40 pN to invade the host cell^8^. Sequence comparisons indicate that an N-terminal extension is also present in *Toxoplasma gondii* myosin A (TgMyoA), and phosphomimetics in that region increased in vitro motility speeds, suggesting that the tunable mechanism is likely conserved amongst the phylum of Apicomplexan parasites^9^.

Actin is one of the most conserved proteins amongst all eukaryotes. Despite being one of the most divergent actins, PfAct1 shows 82% sequence identity with cytoplasmic γ-actin. Given the dynamic nature of PfAct1 filaments, jasplakinolide (JAS), a cycle peptide from the sea sponge *Jaspis johnstoni* that is able to induce actin polymerization, has to be used to stabilize PfAct1 filaments in structural investigations^10,11^. Not surprisingly, monomeric and JAS-stabilized filamentous PfAct1 exhibits features similar to mammalian monomeric G-actin and filamentous F-actin^10,11^. Sequence divergence is primarily located at the binding interface of actin-binding proteins and the nucleotide-binding pocket^10–12^. Furthermore, the contact sites important for filament formation contain notable sequence deviations, particularly within the DNase-1–binding loop (D-loop) which is essential for polymerization^10^. These sequence differences result in a critical concentration for assembly (in the absence of JAS) that is an order of magnitude higher than for skeletal muscle actin filaments or JAS-stabilized PfAct1^13^. Moreover, PfAct1 filaments are highly unstable once ATP has been hydrolyzed and readily depolymerize^13^, likely explaining why these filaments have been difficult to visualize in vivo.

The actomyosin complex is recalcitrant to crystallization. Before 2016, the description of this interface by electron microscopy was limited to 7 Å resolution^14–17^.The recent improvement in electron cryomicroscopy (cryo-EM) technology and software permitted five mammalian actomyosin structures to be solved at a resolution of better than 5 Å: for human non-muscle myosin 2c (NM2c) in the Rigor state at 3.9 Å resolution^18^; for porcine myosin VI (Myo6) in the Rigor state (4.6 Å)^19^; and for rat myosin-Ib (myo1b) in the Rigor (3.9 Å) and in two strong binding ADP (3.3 Å and 3.8 Å) states^20^. All these structures correspond to the states of the motor populated after force generation: the strong ADP state before ADP release, and the nucleotide-free (Rigor) state that have the highest affinity for actin filaments (F-actin) for most myosins. Because the myosin structural elements that bind F-actin are highly divergent in sequence, the actomyosin interface was assumed to vary substantially among myosin motors and is considered hard to predict^19^. This divergence explains why, despite the fact that several structures are available at high-resolution, the structural features underlying F-actin recognition by myosin are still highly debated. A better knowledge of the actomyosin interface is crucial to understand which parts of the interface are general features, i.e. common to all myosins, and which ones are specific to a particular myosin. In addition, which parts of the interface are attributable to define a specific cellular function is currently unknown.

To understand the mechanism underlying force production by an atypical actomyosin complex and how this interaction differs from previously described recognition of F-actin by mammalian myosins^21^, we determined the cryo-EM structure of PfMyoA bound to filamentous PfAct1 stabilized with JAS. Using likelihood-based reconstruction of helical specimens and a real-space modeling approach aided by molecular dynamics for the refinement of the model, we present a structure at a resolution of 3.8 Å. The PfActomyosin interface highlights the conserved features that provide the structural basis for the ability of PfMyoA to move both PfAct1 and mammalian actin filaments at similar speeds. Moreover, comparison of this interface with the mammalian ones described previously allows us to subdivide the actomyosin interface into two regions: (i) the conserved core interface that governs filament recognition for all myosins and (ii) the variable ancillary interface that defines the orientation of the motor on the track and tunes specific motor functions.

## Results

### High-resolution structure of the PfAct1/PfMyoA complex in the Rigor state

Unlike canonical skeletal muscle actin, PfAct1 filaments are dynamic and require a very high critical concentration (~4 µM) to polymerize^13^. Addition of JAS stabilizes PfAct1 filament dynamics and decreases the critical concentration to the nanomolar range^11^, which minimizes the presence of actin monomers in the background of cryo-EM preparations^10–12,22^. Indeed, a 3.8-Å structure of JAS-stabilized PfAct1 filaments has been reported^8^, while a reconstruction of unstabilized PfAct1 filaments has yet to be accomplished.

**Fig. 1:**
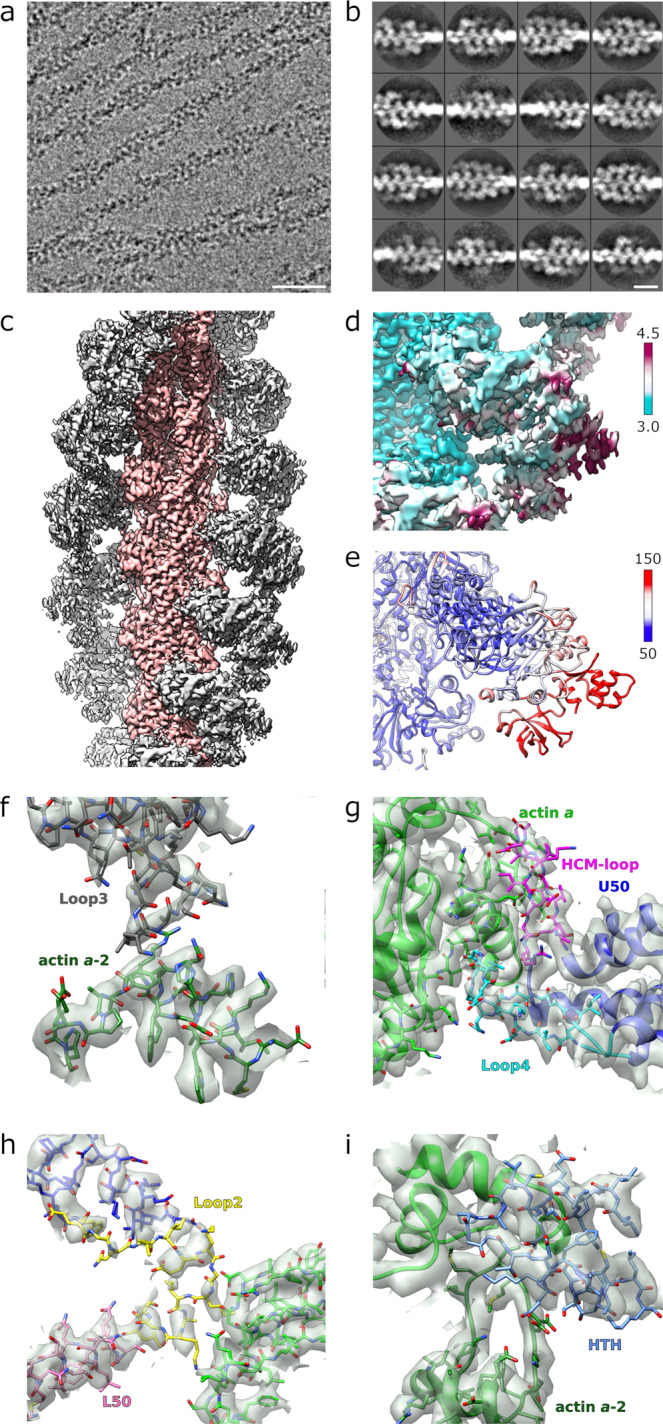
Structure of the PfMyoA motor domain/PfAct1 filament assembly. **a** Representative micrograph (out of 6073) of JAS-stabilized PfAct1 filaments with bound PfMyoA motor domain. The scale bar is 50 nm. **b** Representative classes (out of 1534) from reference-free two-dimensional classification of segments showing lack of cooperative binding (full decoration). The scale bar is 20 nm. **c** Reconstruction of JAS-stabilized PfAct1 filaments (pink) with bound PfMyoA motor domain (gray). The pointed (-) end of the filament is up. **d** Local resolution mapped on the reconstruction. Values of color bar in Å. **e** Atomic temperature factors mapped on the refined model. Values of color bar in Å^2^ **f**–**i** Density maps and atomic model for all the actin binding elements of PfMyoA at the actomyosin interface. All the different actin binding elements are colored differently: The hypertrophic cardiomyopathy loop (HCM) in pink; Loop2 in yellow; Loop3 in dark gray; helix-turn-helix (HTH) in dark blue (see also Supplementary Fig. 1).

To identify experimental conditions for high-resolution structure determination of the *Plasmodium falciparum* actomyosin complex, various PfMyoA constructs were used, including the full-length protein and the motor domain. However, only sparse and non-cooperative binding to both rabbit skeletal and JAS-stabilized PfAct1 filaments was observed for all pairs. This lack of cooperativity contrasts with the highly cooperative nature of essentially all other previously reported myosin motors binding to actin filaments, which appear either fully decorated or as bare filament stretches in the same field of view in cryo-EM preparations, significantly assisting in particle selection and image processing. Actin-activated ATPase assays showed that the phosphomimetic mutation T417D located in the hypertrophic cardiomyopathy (HCM) loop of the PfMyoA heavy chain (at the TEDS site)^23^ resulted in a ~3-fold enhancement in actin binding affinity while V_max_ is unchanged^24^. The use of a PfMyoA motor-domain construct with a T417D mutation increased occupancy of binding to JAS-stabilized PfAct1 filaments in cryo-EM preparations (Fig. 1a), although even with the improved occupancy, PfMyoA binding remains sparse (Fig. 1b).

Using these conditions, cryo-EM and Bayesian analysis of helical specimens^25^ were employed to determine a 3.8 Å cryo-EM reconstruction of the complex (Fig. 1c, Supplementary Fig. 1). The reconstruction shows the converter in the post power-stroke position, similar to the position obtained for actin bound myosin II in the Rigor state^18^. Using the atomic models of the PfAct1^11^ and PfMyoA in the Rigor-like state^8^ as starting points, we were able to accurately place the protein chains throughout most of the density map and, in regions with higher resolution, to confidently discriminate and place side chains (statistics in Supplementary Table 1).

**Fig. 2:**
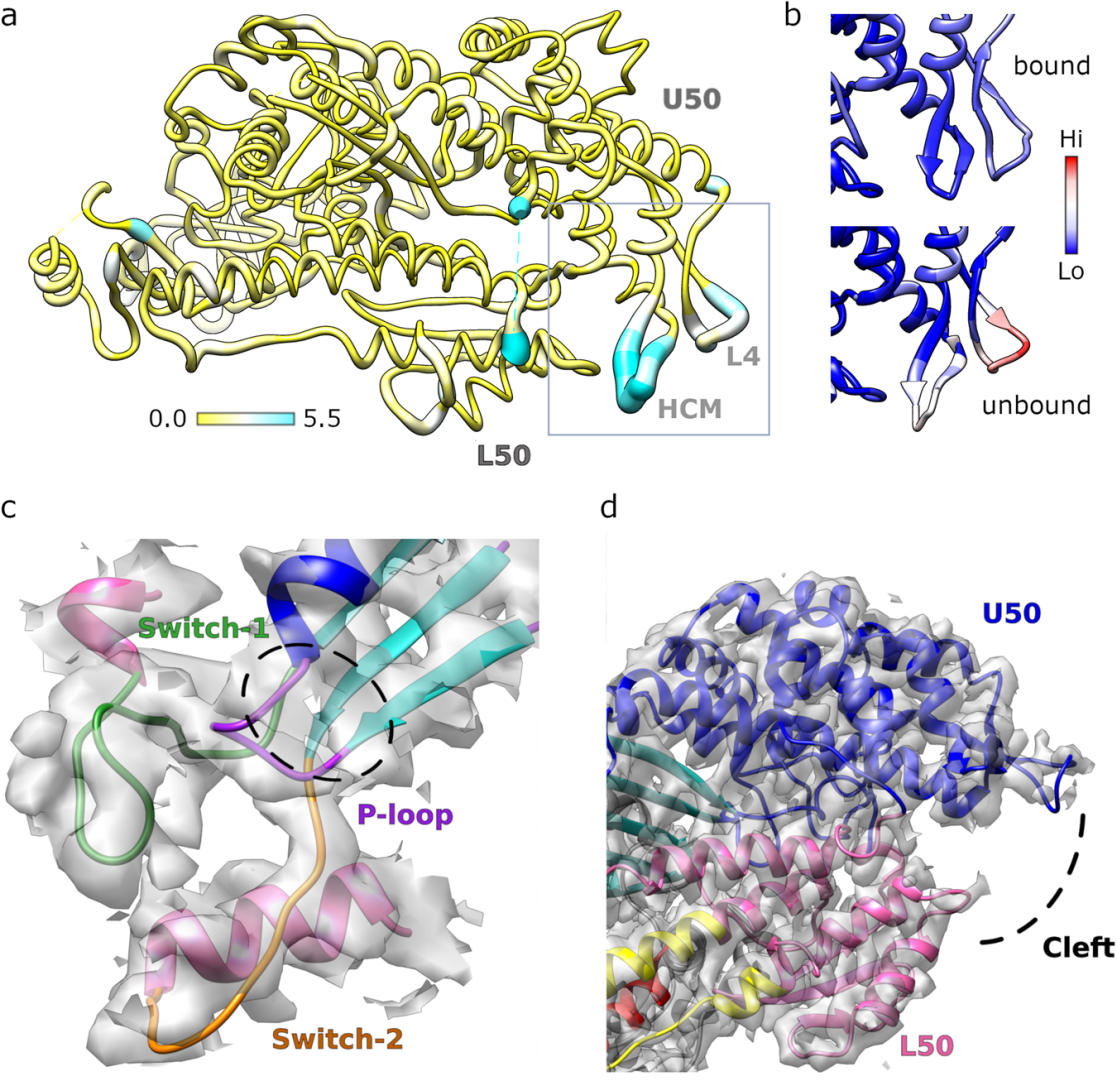
Structure of the PfAct1-bound PfMyoA motor. **a** Atomic model of actin-bound PfMyoA motor domain with root mean square differences to the rigor-like unbound structure mapped in thickness and color, from 0–5.5 Å. Only residues present in both structures are shown. The hypertrophic cardiomyopathy loop (HCM), loop 4 (L4) and the upper and lower 50 kDa domains (U50, L50) are labeled. **b** Comparison of the normalized atomic temperature factors between the bound (upper panel) and unbound (lower panel) structures. The region shown corresponds to the box in A. **c** Density map and atomic model in the active site of PfMyoA shows without ambiguity the absence of nucleotide. There is no density in the cavity (black dashed lines) composed by the Switch-1 (green), Switch-2 (orange) and the P-loop (purple). **d** The density map and atomic model of the actin binding cleft located between the upper 50 kDa (U50, in blue) and the lower 50 kDa (L50, pink) domains.

Regions of the motor further away from the PfAct1 filament such as the Converter and a part of the N-terminal PfMyoA subdomain are less well defined (Fig. 1d) in the reconstruction but this is readily explained by increased atomic temperature factors in those regions (Fig. 1e). In fact, the model displays a characteristic repartition of the atomic temperature factors: the lowest atomic temperature factors are located close to the PfAct1/PfMyoA interface for the two motor subdomains (Upper-50 kDa and Lower-50 kDa) involved in binding the PfAct1 filament. Higher atomic temperature factors are located in the N-terminus (N-term extension and the SH3-like structural domain), the Transducer, the Relay and the Converter. The regions that display the highest atomic temperature factors are located in the Converter. This suggests a certain flexibility of the Rigor conformation of PfMyoA in this region, as previously observed for other actomyosin reconstructions^17^. Actin and the actomyosin interface are well defined in the electron density (Fig. 1f–i). The tip of Loop2 (residues 632-635) is partially disordered (Fig. 2h), but the interactions with actin are visualized without ambiguity.

### Rigor complex formation occurs with only minor conformational changes to PfMyoA

Despite the availability of several high-resolution cryo-EM studies^18–20^, one of the most debated points has been the conformational changes induced by the formation of the actomyosin assembly both in the actin filament and in the myosin motor. The PfMyoA motor bound to the PfAct1 filament closely resembles the crystal structure of the Rigor-like conformation^8^ with overall root-mean-square displacement (rmsd) of 0.84 Å. (Fig. 2a, b), There is no electron density at the active site of PfMyoA in this reconstruction that can be attributed to a nucleotide (Fig. 2c). This structure thus represents the Rigor state at the end of the powerstroke in which no nucleotide is bound to the motor domain and, like Rigor structures of other myosins, PfMyoA adopts an inner cleft closure in this state (Fig. 2d). The degree of cleft closure for the Rigor state of PfMyoA is similar to that of the Rigor states of other myosins^18–20^.

Not surprisingly, some actin-binding elements, namely the HCM-loop (407–430) and Loop4 (363–387) differ in conformation between the PfAct1-bound Rigor and Rigor-like crystal structures of PfMyoA. Note that these two actin-binding elements are quite flexible for the unbound motor but become restrained when myosin binds actin (Fig. 2b). As in the Rigor-like crystal structure, the Rigor conformation of the PfMyoA motor is stabilized by a number of atypical interactions involving several elements implicated in mechanical communication during the power stroke as well as the PfMyoA specific N-terminal extension, which is a key element of the unique tunable mechanism of force-production of PfMyoA via its phosphorylation state^8^.

### Complex formation does not shift the D-loop of PfAct1 but increases the helical twist

**Fig. 3:**
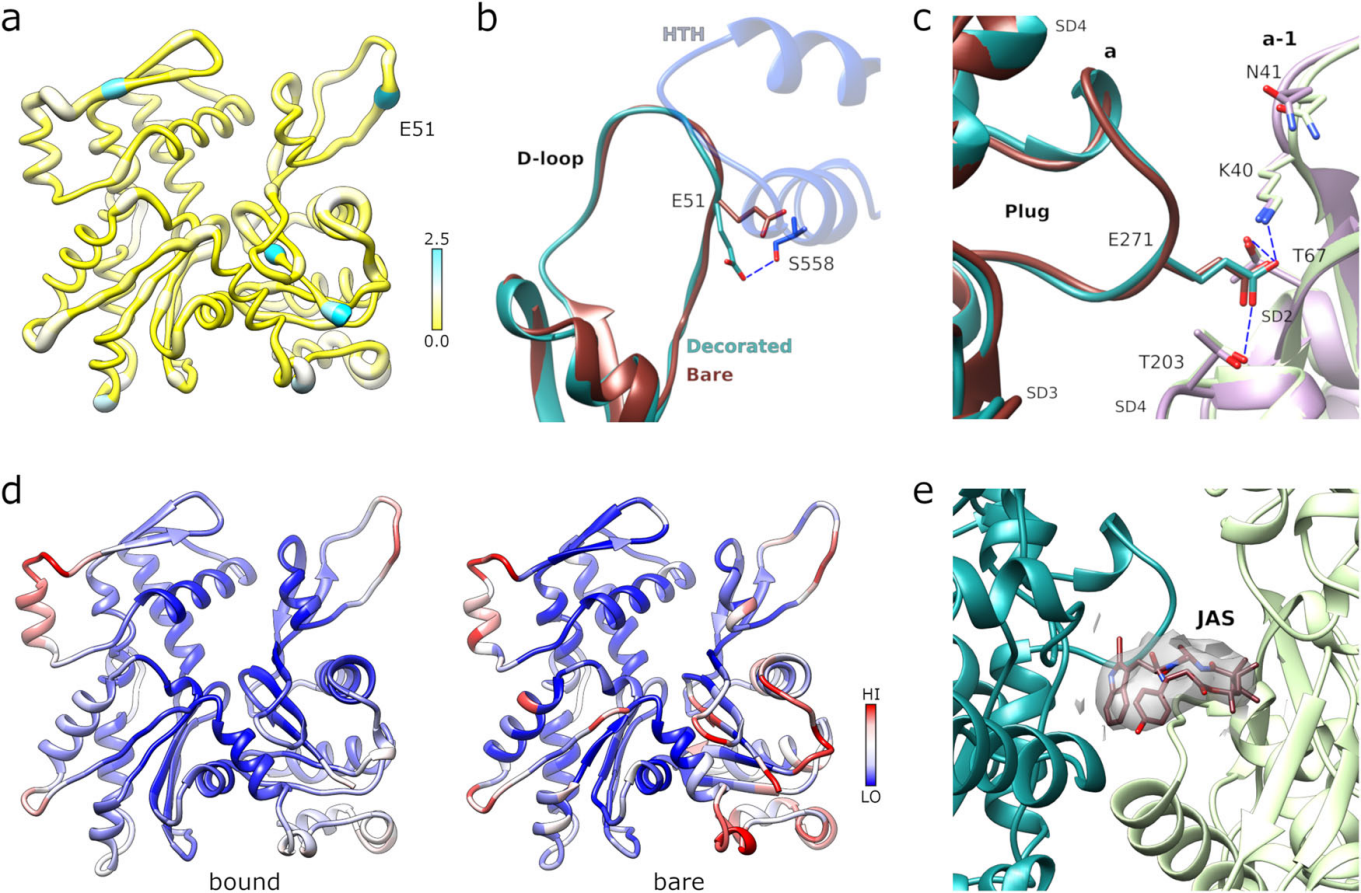
Structure of the PfMyoA-bound PfAct1 filament. **a** Atomic model of filamentous PfAct1 while bound to PfMyoA with root mean square differences to the bare PfAct1 filament structure mapped in thickness and color, from 0–2.5 Å. **b** The D-loop shows only minor structural differences in the bound (cyan) compared to the bare (chocolate red) filament (PDB code 5OGW; https://www.rcsb.org/structure/5OGW)^11^ with the largest difference occurring at residue E51. In the decorated filament, the conformation of the sidechain of E51 is reoriented in order to establish a polar contact of with S558 from the HTH of PfMyoA (dark blue). **c** The plug conformation is identical in both structures and the specific polar interactions are conserved. **d** Comparison of the normalized atomic temperature factors between the bound (left) and unbound (right) PfAct1structures. **e** Jasplakinolide (JAS) is well defined in the density map of the PfMyoA bound PfAct1 structure.

The binding of PfMyoA to the PfAct1 filament also does not induce major changes in the conformation of the PfAct1 filament. The structures of the bare^11^ and decorated PfAct1 filaments superimpose with an rmsd of 0.67 Å (Fig. 3a). Within the interface, only the D-loop shows some modest changes (~1.4 Å maximum between Cα positions) for residues 51–55 that interact with PfMyoA. Notably, the re-orientation of the side chain of E51 is required to avoid a steric clash with myosin (Fig. 3b).

The helical parameters are changed slightly from the unbound PfAct1 filament with an essentially identical subunit rise of 27.3 Å and a helical twist of −168.1°, a change in twist from unbound PfAct1 filaments (−167.5°)^11^ by 0.6°, a change similar to that observed for actin binding of human myosin 2 C and chicken myosin V^18,26^. Despite this change in helical twist, neither the intra- and inter-strand interactions nor the specific polar bonds established by the plug are visibly altered in the decorated structure (Fig. 3c). Thus, as in the unbound structure^11^, these interfaces are generally conserved with those in canonical actin and the key differences explaining the intrinsically decreased stability of PfAct1 filaments^11^, are still present in the PfMyoA bound filament.

There is a substantial stabilization of PfAct1 when comparing the normalized relative atomic temperature factor distributions of decorated and undecorated PfAct1 in the interface region (Fig. 3d). Surprisingly, the D-loop is the only part of the interface that does not show relative stabilization. The D-loop is one of the few regions that differ in structure in the PfAct1 filament compared with canonical actin. The conformations of the PfAct1 D-loop in the bare or PfMyoA decorated structures are both shifted outward by about 1–2 Å in relation to that of canonical F-actin^11^. The JAS peptide (Fig. 3e) and the ADP nucleotide (Supplementary Fig. 2) are seen without ambiguity in the electron density at the same positions as in the undecorated filament.

It appears that the PfAct1 D-loop conformation is less sensitive to additional factors binding to the filament than mammalian D-loops. For example, JAS stabilization of rabbit skeletal actin filaments induces an open conformation of the D-loop^27,28^, while JAS-stabilized PfAct1 filaments adopt the closed conformation that most high-resolution structures of canonical actin filament structures adopt^11,27,29,30^. Similarly, binding of NM2c and Myo6 induces a shift in the D-loop position towards myosin in the corresponding actin subunit^18,19^ while there is no perceivable shift in the PfAct1 D-loop upon PfMyoA binding. Sequence differences between PfAct1 and canonical actin substantially alter the intra-strand interface and the conformation of the D-loop. While in canonical F-actin, Q50 plays a critical role by forming a N-H…π bond with Y170^29^, the equivalent residue in PfAct1 is glutamate E49 that adopts an opposite orientation.

This polymorphism could also explain why the open conformation is not observed in JAS-stabilized PfAct1 filaments, although it would be compatible with the PfMyoA motor domain positioning when bound to PfAct1. The open conformation results from a sharp bend that occurs in the D-loop at residue Q43. Because the equivalent residue is proline P42 in PfAct1, the conformation of the D-loop is restricted, and the open conformation cannot be explored. The open conformation of the D-loop in other actins has been proposed to increase the stability of the actin filament^27,28,31^. The fact that this conformation cannot be adopted by the D-loop of PfAct1 would thus contribute to the instability of PfAct1 filaments.

### The actomyosin Rigor interface displays a conserved actin footprint

**Fig. 4:**
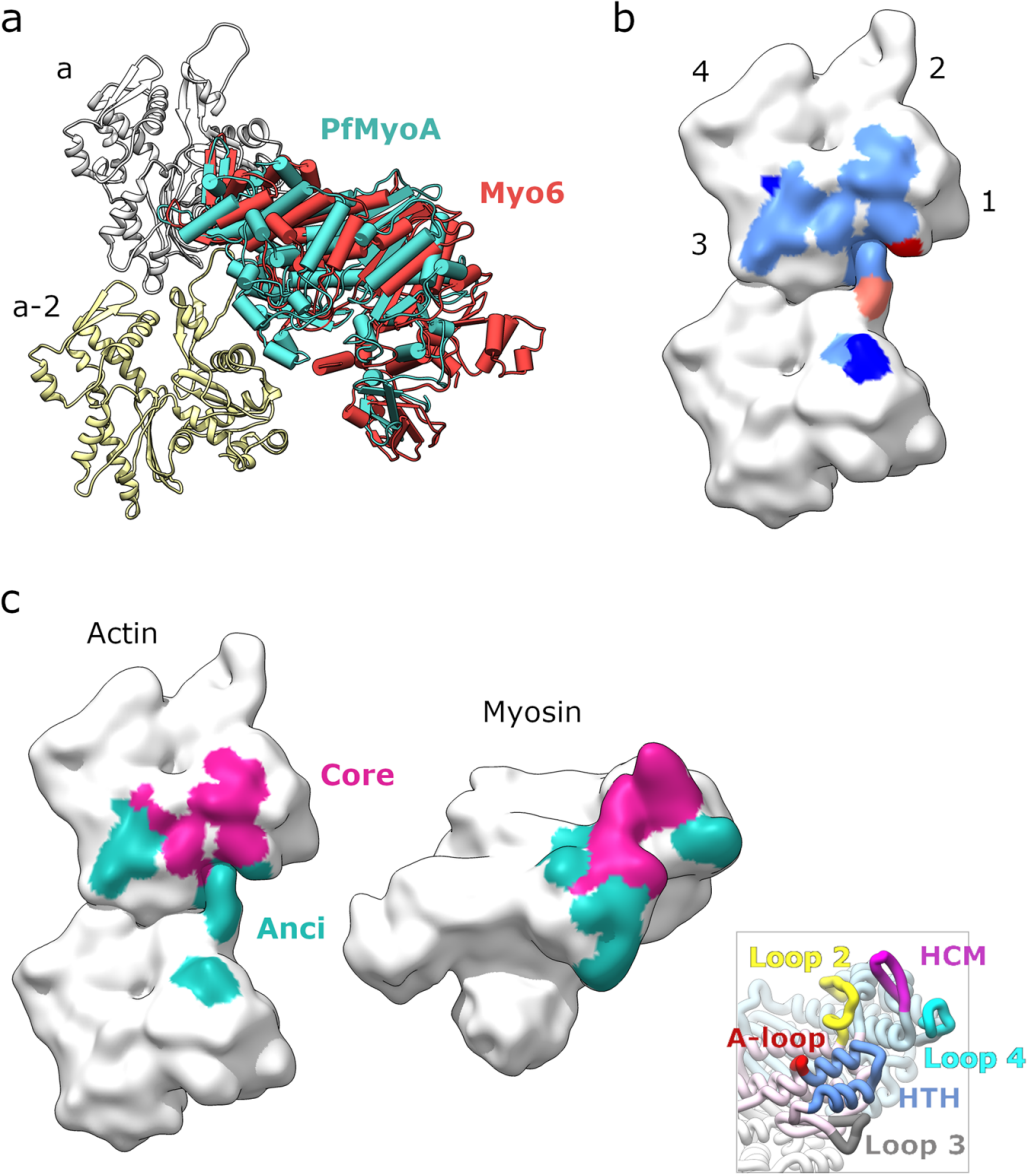
Actomyosin interaction footprint analysis. **a** Overlay of the filament bound structures of PfMyoA (teal) and Myosin 6 (Myo6; red) revealing very similar orientations in relation to the filament. **b** Mapping of myosin footprints onto the actin filament surface. Two longitudinal neighboring actin monomers are shown. The subdomains are labeled in the upper monomer. The light blue region is contacted by all four myosins. The dark blue region is contacted by all myosins except for PfMyoA. The orange region is contacted by Myo1b and NM2c, the red region is contacted by NM2c only. **c** Mapping of the core (magenta) and ancillary (Anci, teal) interfaces as defined by conservation of interactions (see Supplementary Table 2) onto an open-book representation with the actin-filament surface on the left and the myosin surface on the right. The inset in the lower right corner shows the primary myosin structural elements partaking in interactions in the same orientation (HCM: hypertrophic cardiomyopathy loop; HTH: helix-turn-helix motif; A-loop: activation loop).

Among the Rigor structures solved to date at high resolution, Myo1b and NM2c share a similar overall orientation of the motor domain on the filament, while the Myo6 Rigor structure differs significantly in the overall orientation^21^. Interestingly, comparison with these structures indicates that PfMyoA adopts an overall orientation that is most similar to that of Myo6 (Fig. 4a). Thus, the difference in overall orientation previously observed for Myo6 compared to other motors^19^ is not related to the motor directionality. This observation raises the question of what dictates the differences in positioning of the motor and what are the exact interactions formed at the actomyosin interface that govern the orientation. Both PfAct1 and PfMyoA diverge significantly from the sequences of their mammalian counterparts, raising the question of whether the Rigor interactions between PfMyoA and PfAct1 differ from those previously described due to sequence adaptation co-evolution.

To study the actomyosin interface in the PfAct1/PfMyoA Rigor structure, the footprint of the myosin on the PfAct1 filament was determined (Fig. 4b, c). Similar to other myosins^21^ PfMyoA interacts with the filament through five canonical structural elements. These are the HCM-loop (407-430); Loop2 (625-643); Loop3 (566-580); Loop4 (363-387) and the Helix-Turn-Helix (HTH, 540-565). The Activation Loop (A-loop) is also considered a canonical actin binding element and has been reported to be a minor part of the Rigor interface of NM2c and Myo1b^18,20^. In PfAct1/PfMyoA, the activation Loop (524-539) is not part of the Rigor interface, as is also the case for Myo6^19^. The mean actin/myosin interaction area of all high-resolution Rigor actomyosin structures is 1607.5 Å^2^ with a standard deviation of 58.1 Å^2^. Thus, the size of the interface is quite well preserved. Interestingly, almost all the PfAct1 residues that interact with PfMyoA are conserved compared to canonical actins (Supplementary Table 2; Supplementary Fig. 4). The only region of divergence in this footprint is the D-loop. Despite the divergence in sequence of the actomyosin system found in the apicomplexan glideosome, the PfMyoA residues that are part of the interface define a conserved actin footprint.

### The footprint is defined by the recognition of a conserved central core interface with less extensive contributions from a more peripheral ancillary interface

The interactions establishing contacts between actin and myosin in the PfAct1/PfMyoA structure were compared to those of other Rigor structures to deduce how the divergent actin-binding loops of distinct myosins accommodate binding to this conserved actin footprint. The conservation of the residues involved in the actomyosin interface has been a subject of debate. Few actomyosin structures are available at a sufficient resolution to confidently deduce the position of residues and interactions made at the interface, and thus a structure fully describing side chain interactions and solvent contribution is still unavailable. However, sufficient differences in main chain loop interactions and some of the side chains defined in cryo-EM maps provide enough information to describe the main features of the Rigor interfaces (PfAct1/PfMyoA described here and in references^18–20^). The study carried out with Myo6 highlighted the divergence in the interface of this motor compared to previously described actomyosin Rigor complexes^19^ whereas the study performed on Myo1b described only minor differences with the NM2c interface^20^. The PfAct1/PfMyoA interface was compared to those of Rigor structures of NM2c, Myo1b and Myo6 after aligning the structures using the F-actin region involved in myosin recognition. This analysis allowed us to distinguish between a large core interface that is common among all four myosins, and a more divergent and ancillary interface (Fig. 4b, c). The core interface, which is essentially completely conserved, is established by the HCM-Loop and most interactions of the HTH, extended by conserved contributions of Loop2. The smaller ancillary interface which is more divergent and established by the HS helix of the HTH, Loop3, Loop4 and, in some cases, additional contributions from the Activation Loop and the strut (Supplementary Table 2). A few additional interactions from Loop 2 and the HW helix may also complete the ancillary interface for some Rigor structures. Interestingly, despite sequence divergence of PfAct1 compared to canonical actin, PfMyoA binds to the actin surface where the sequence is conserved in both actins (Supplementary Table 2). Only two polymorphisms are found in actin and they are located in the ancillary interface. They correspond to a residue that interacts with Loop4 (^Pf^V330, ^Sk^I331) with no major impact on the interface and to a sequence difference in the D-loop (^Pf^E49, ^Sk^G50).

### The conserved core interface of Rigor actomyosin structures

The conserved core interface of the Rigor state corresponds to the largest part of the interface, including interactions on either side of the myosin inner cleft with the interface between the D-loop and subdomains 1 and 3 (SD1, SD3) of the filament (Fig. 4b; Supplementary Table 2A). Most of this interface corresponds to extensive apolar contacts with few specific interactions. Several apolar interactions occur from residues of the HR helix or the turn of the HTH. While the sequence of these residues varies amongst the four myosins, the regions of interaction on the actin filament are conserved. The HTH structural element, the actin binding element most conserved in sequence, comprises two invariably conserved residues that interact with actin in a conserved fashion in all Rigor structures. The first conserved residue is an acidic amino-acid from the HR-helix (D544 in PfMyoA) that establishes polar interactions with a serine of SD1 (S351 in PfAct1, S350 in canonical actin). The second conserved residue is a proline at the tip region of the HTH (P549 in PfMyoA) that plugs into a hydrophobic cavity at the D-loop/SD1 interface between the two neighboring actin monomers. The main actin residues recognizing this HTH turn region belong to SD1 and SD3, close to subdomain 2 (SD2) of the actin below. While less preserved amongst the Rigor structures, residues on either side of this proline in the HTH turn indeed provide additional hydrophobic interactions in the cavity in a lock-and-key fashion (Supplementary Table 2A, Supplementary Fig. 3).

The second part of the core interface involves the HCM-loop, which is a β-hairpin that presents hydrophobic residues that establish contacts with actin via residues lining the cleft between SD1/SD3 on the hydrophobic cleft (Supplementary Table 2A). The number of the HCM-loop residues that interact with F-actin and their sequence have been reported to be variable among myosins^19,20^. While the PfMyoA HCM-loop residues that contact the PfAct1 filament do not correspond to either of the other myosin sequences, they anchor the HCM-loop in a similar orientation on the actin surface. However, the variability in the nature of these residues greatly influences the exact interface formed. For example, the same negative actin surface residues (E335 in PfAct1, E336 in canonical actin) can either form polar interactions with the first strand of the HCM-loop β-hairpin (PfMyoA, NM2c, Myo1b) or the second strand (Myo6) (Supplementary Table 2A). Another example is the TEDS residue, a consensus site for phosphorylation^23^ found at the tip of the β-turn, which is either a negatively charged residue (E,D) or one that can be phosphorylated (T,S). In the Rigor structures, this residue only interacts with a positively charged residue on actin when the position is occupied by a glutamate (i.e. Myo1b or the PfMyoA T417D mutant used in this study). Otherwise, there is no interaction of this residue with actin at the Rigor interface.

Lastly, the C-terminal end of Loop2 complements the interaction in the core surface by interacting with a negatively charged patch on SD1 in between the HCM-loop and HTH-turn interfaces (Supplementary Table 2A). Loop2 is the most variable region of the actin binding surface found on myosins—both in size and sequence. Despite this variability, Loop2 binds essentially to the same surface on the SD1 of F-actin and is usually stabilized when binding to F-actin^16^. As it is the case for Myo1b, PfMyoA has a short Loop2. One common feature among the Myo1b and PfMyoA Rigor complexes is that the interactions formed involve positively charged residues of Loop2, extended to positively charged residues of the strut (Supplementary Table 2A). Although these residues are not conserved in their position (G633 and K634 in PfMyoA), they interact with the same residues of the actin surface (G24, D25, D26 in PfAct1).

### The ancillary interface of the Rigor structures

The ancillary interface (Fig. 4c, Supplementary Table 2B) comprises the most variable interactions that are also regions of the interface distal from the main core. This actin-binding surface is composed of residues interacting with the HS helix of the HTH, Loop3, Loop4 and, in some cases, the Activation Loop. The N-terminus of the actin subunits is flexible and often disordered in the Rigor structures, but it may participate in this ancillary interface. This is clearly indicated in the cryo-EM map of the NM2c Rigor structure in which contacts with actin’s N-terminal residue E5 occur via residues of loop2 and the HW helix (Supplementary Table 2B; Supplementary Fig. 5A; Supplementary Fig. 4). All four sites of the ancillary interface differ in the nature of the contacts made among actomyosin structures (Supplementary Fig. 3). (i) Loop3 is highly variable in sequence and in length, but residues of the N-terminal part of its sequence bind the same residues in SD1 although the exact contacts made for the different Rigor myosin interfaces are different (Supplementary Fig. 5B). (ii) Loop4 is also highly variable in sequence and in conformation and has fewer and non-conserved contributions compared to elements that form the core interface (Supplementary Fig. 5C; Supplementary Table 2B). The tip of the loop interacts with SD3 of actin. In the NM2c Rigor complex, there are only minor contacts with SD3 and Loop4 and this region favors interactions with tropomyosin^18^. It is tempting to speculate that the contribution of Loop4 to binding of the filament is class-dependent and allows specificity in the recognition of the target actin filament as well as tropomyosin interactions. Myo1b is indeed less efficient on actin-tropomyosin filaments and this contributes to the selective localization of this myosin in cells^32,33^. (iii) The Activation Loop is not part of the Rigor interface of PfAct1/PfMyoA and that of Myo6. Some contributions of this loop to the Rigor interface occur for NM2c and Myo1b although these are relatively minor (Supplementary Table 2B). (iv) Lastly, the HS helix of the HTH motif provides critical interactions with the D-loop and thus can extend the interface close to the core (Supplementary Fig. 5D; Supplementary Fig. 3). In the PfAct1 and canonical actin structures, the D-loop is the structural element that explores the most dynamic structural conformations; and these are influenced by the nucleotide-state of the filament^27,28^. PfAct1 is divergent from canonical actin in the D-loop conformation and this is also where the main sequence differences of the footprint reside, i.e. the presence of a glutamate (E49) instead of a glycine (G50) found in canonical actin. Interestingly, while most myosins have a negatively charged residue on the HS helix facing G50, the sequence of PfMyoA is a serine (S558) at this position, avoiding some charge repulsion between the residues in contact. Myo6 has also a serine in this position and the conformation described for the D-loop locally differs from that of Myo1b and NM2c, allowing it to optimize contacts with the small serine side chain (Supplementary Table 2B). This D-loop conformation found for a canonical F-actin when bound to Myo6, would not be compatible with the presence in the myosin HS helix sequence of an aspartate or a glutamate. We conclude that the interactions of the HS helix with the D-loop can cause local and possibly important induced fits when the motor and the actin filament interact. This region of the ancillary interface close to the core interface is likely to play an important role in defining how different myosins would be more or less efficient depending on the age of the F-actin filament they are interacting with^34^.

### The ancillary interface determines the position of myosin on F-actin in the absence of nucleotide

The motor positioning on the filament can vary greatly when the cryo-EM Rigor structures are compared. The core interactions are at the center of the interface and are not likely to influence this positioning significantly. In contrast, the ancillary interface probably plays a more important role in positioning. The fact that the lower 50 kDa domain differs in its positioning for PfMyoA compared to the other motors, is likely linked to the interactions that occur between the HS helix and the D-loop, which themselves depend on the conformation and sequence of the D-loop. In the case of Myo6, for which the difference in the lower 50 kDa domain orientation is greater, optimization of interactions with Loop3 is likely to be a key factor.

A closer look at the interface indicates how the core interactions can be conserved despite a rather large difference in the position of the different motors on F-actin. Local adaptation in the conformation of the actin residues near the C-terminus is also likely to optimize the core interactions with the HR-helix. Another key element that may conserve the core interactions involves flexibility in the HCM-loop, which optimizes the orientation of the upper 50 kDa domain to accommodate ancillary interactions with Loop4. The flexibility at the beginning and end of both the HCM-loop and Loop4 are restrained but could be essential for allowing the repositioning of the upper 50 kDa domain resulting in conservation of the footprint and the cleft closure in these different Rigor structures.

### Motility assays show similar speeds with various actin and myosin pairs

The finding that canonical actomyosin systems and the divergent *P. falciparum* actomyosin system share a common interaction footprint was unexpected. To test whether this equivalence in footprints translates to equivalence in functionality, motility assays were performed using PfMyoA and smooth muscle myosin paired with either skeletal muscle actin, smooth muscle actin, or PfAct1 filaments. To allow meaningful comparison, identical setups were used for evaluating motility, i.e. the respective myosin motors were adhered to the coverslip and the speed of unrestrained actin filaments being moved was evaluated. This setup should be insensitive to the differences in twist between canonical actin and PfAct1 filaments.

Interestingly, no difference in speeds were observed when PfMyoA moved JAS-stabilized PfAct1 filaments, skeletal muscle actin or smooth muscle actin. Although JAS has profound effects on the polymerization properties of PfAct1^13^, the PfMyoA motor did not discern a difference when it moved JAS-stabilized versus unstabilized PfAct1 filaments. Likewise, when smooth muscle myosin was paired with JAS-stabilized PfAct1 filaments, skeletal muscle actin, or smooth muscle actin, no difference in speeds were observed (Table 1). These assays demonstrate that PfMyoA moves all actin filaments with essentially identical speed, and that the conventional motor smooth muscle myosin also moves PfAct1 filaments at indistinguishable speeds to how it moves canonical actin filaments. Similarities in the interaction footprint thus translate directly into similarities in motility speeds.

**Table 1:** In vitro motility speeds of different actin and myosin pairs.

Actin	PfMyoA (µm/s)	Smooth muscle myosin (µm/s)
PfAct1/JAS	3.55 ± 0.53 (*n*=905)	0.53 ± 0.19 (*n*=1441)
PfAct1	3.98 ± 0.67 (*n*=1545)	n.d.
Skeletal	3.85 ± 0.59 (*n*=942)	0.62 ± 0.29 (*n*=953)
Smooth	4.21 ± 0.63 (*n*=1232)	0.51 ± 0.22 (*n*=1019)

Values are mean ± SD. Two separate experiments were performed for each condition. Each pairwise combination of a given motor with different actins is statistically insignificant using a two-sided *z-*test. All PfMyoA/actin pairs, *p* ≥ 0.416; all smooth muscle myosin/actin pairs, *p* ≥ 0.774 (PfMyoA/PfAct1/JAS vs. PfMyoA/PfAct1: *p* = 0.604; PfMyoA/PfAct1/JAS vs. PfMyoA/skeletal: *p* = 0.704; PfMyoA/PfAct1/JAS vs. PfMyoA/smooth: *p* = 0.416; PfMyoA/PfAct1 vs. PfMyoA/skeletal: *p* = 0.875; PfMyoA/PfAct1 vs. PfMyoA/smooth: *p* = 0.803; PfMyoA/skeletal vs. PfMyoA/smooth: *p* = 0.673; Smooth muscle myosin/PfAct1/JAS vs. Smooth muscle myosin/skeletal: *p* = 0.799; Smooth muscle myosin/PfAct1/JAS vs. Smooth muscle myosin/smooth: *p* = 0.954; Smooth muscle myosin/skeletal vs. Smooth muscle myosin/smooth; *p* = 0.774).

## Discussion

The binding of PfMyoA to the PfAct1 filament does not induce major conformational changes in the actin filament or in the motor. The cleft closure in the motor is similar to that found for other myosin motors bound to actin^18–20^ and the Rigor-like crystal structure of PfMyoA^8^. The D-loop in the bound PfAct1 filament is quite similar to the conformation seen in other Rigor structures, all of which have been reported to have a closed D-loop conformation, consistent with the fact that the motor was bound to ADP F-actin filaments^18–20,35,36^. The D-loop conformation in PfAct1 filaments does not shift position upon PfMyoA binding, which differs from at least two mammalian actomyosins, namely NM2c and Myo6 in the Rigor state^18,19^.

The actomyosin interface is composed of a highly conserved actin core extended by more variable ancillary regions. Comparison of currently available high-resolution cryo-EM reconstructions of myosin strongly bound to actin filaments provides insights about the conserved elements in the strong binding states of myosin motors: (i) a conserved motor domain conformation with a specific cleft closure, (ii) a conserved surface on the bound actin filament that participates in interaction with myosin and (iii) an absence of major conformational changes of the filament when binding a motor, although a relative decrease in the dynamics of some filament regions occurs.

Remarkably, the divergence in sequence of PfAct1 compared to canonical actin does not impact residues of the footprint, with the notable exception of the D-loop, which is variable in sequence. Thus, the myosin side of the interface remarkably adapts to recognize the same residues of the footprint, by forming locally distinct interactions. This makes the interface variable in detail, as previously observed^19,20^, while retaining conserved general principles. Local adaptations on myosin lead to recognition of the same residues on the actin surface for the different myosins. Motility assays support these structural observations by showing that a given myosin motor can move heterologous actins at the same speed as its cognate actin.

The remarkable finding that there are conserved features among Rigor structures spanning the phylogenetic tree from apicomplexans all the way to humans is important for understanding how motors have evolved. Myosins are traditionally classified as members of a superfamily based on phylogenetic analysis of their head domains^37^. While these types of phylogenetic relationships provide insights into how specific myosin functions are related within and between families, the evolutionary history of myosin is not revealed by such studies. This history can only be assessed by considering the evolution of entire organisms. Except for the structure presented here, all other high-resolution actomyosin structures solved up to now were mammalian.

Myosin is specific to Eukaryotes and does not occur in Bacteria or Archaea. The phylogenic relationship between *P. falciparum* and mammals is about as distant as it can be within the eukaryotic branch of the phylogenetic tree of life. In the latest super-group model of the tree^38^, the branch that contains the TSAR (Telonemids, Stramenopiles, Alveolates, and Rhizaria) super-group, which includes *P. falciparum* (Alveolata), and the one that contains mammals (Amorphea), divide very near the root of the tree. While the exact position of the root is still under debate, it is clear that the common ancestor of *P. falciparum* and mammals is closely related to the Last Eukaryote Common Ancestor (LECA).

Extensive phylogenetic analysis indicates that the LECA had only two different myosins present in its genome^39^. The high-resolution structure of *P. falciparum* actomyosin, in conjunction with the mammalian counterparts, thus gives us the opportunity to infer features of the actomyosin complex and its interactions that were already present in the LECA and have been preserved for over a billion years^40^. The fact that the core actomyosin interactions are essentially identical in all currently known structures, indicates that they have been already present in this form in the LECA and did not change substantially during evolution since then. It is likely that these core interactions are preserved among all actomyosins. The plethora of myosin families and functions thus is independent of this core interaction and is a consequence of secondary actomyosin interactions governed by the divergent portions of the interface as well as other structural elements within specific myosins that are far removed from the interface.

The common, evolutionary conserved interactions that occur for all Rigor structures, provide an anchoring site of the motor on the actin filament that appears to be independent of myosin type, species and function. Importantly, there is also an ancillary footprint on actin with varying actomyosin interactions; the specificity of track recognition (e.g. selection depending on the tropomyosin bound or depending on the age of the filament) resides in the subtlety of the differences found in this interface. In addition, it is likely that these subtle differences play a role in the evolution of cellular roles of specific myosins, since the time spent in the actin-bound states is critical to define how long a motor stays bound to the filament. Current evidence indicates that the internal rearrangements and the stabilization of the Rigor state by interactions within the motor that involve the N-terminal extension can greatly impact ADP release and the duty ratio for both Myo1b and PfMyoA^8,41^. It remains to be explored whether the specific variations among Rigor structures are also associated with properties of the motor such as the duty ratio, which for several myosins depends mainly on the ADP release rate. Defining whether possible local changes at the actin interface can tune ADP release will require further investigation.

## Methods

### Myosin expression and purification

A truncated PfMyoA heavy chain (PlasmoDB ID PF3D7_1342600/ GenBank accession number XM_001350111.1) coding for the motor domain (amino acids 1-K768, PfMD), with a T417D mutation to enhance actin affinity, obtained by PCR using gBlocks gene fragments (Integrated DNA Technologies) with Sf9 cell preferred codons as template. Residue K768 was followed by a Gly-Ser linker and a C-terminal FLAG tag. The PCR product was cloned into the baculovirus transfer vector pAcSG2 (BD Biosciences) to make recombinant virus^24^. The PfMD construct was co-expressed with the PUNC chaperone in Sf9 cells. Cells were grown for 72 hrs, harvested and lysed by sonication in 10 mM imidazole, pH 7.4, 0.2 M NaCl, 1 mM EGTA, 5 mM MgCl_2_, 7% (w/v) sucrose, 2 mM DTT, 0.5 mM 4-(2-aminoethyl) benzenesuflonyl fluoride, 5 µg ml^−1^ leupeptin, 2 mM MgATP. 3 mM MgATP was added prior to a clarifying spin at 200,000 *g* for 40 min. The supernatant was applied to a FLAG-affinity chromatography (Sigma), the column washed with 10 mM imidazole pH 7.4, 0.2 M NaCl, and 1 mM EGTA and the myosin eluted with the same buffer containing 0.1 mg ml^−1^ FLAG peptide. Peak myosin fractions were pooled and concentrated using an Amicon centrifugal filter device (Millipore). PfMD was dialyzed versus10 mM imidazole pH 7.4, 10 mM NaCl, 1 mM MgCl2, 1 mM EGTA, I mM NaN3, 1 mM DTT prior to use.

### Preparation of JAS-stabilized actin filaments

PfAct1 (PlasmoDB PF3D7_1246200/GenBank accession number XM_001350811.1) followed by a linker and human thymosin-β4 (accession number AAI41977.1) was synthesized using gBlocks gene fragments with *Sf*9 cell preferred codons and cloned into pAcUW51 for expression in S*f*9 cells. The initial Cys residue of actin was not included in the construct because it is not present in the mature, processed protein. The thymosin-β4 gene was separated from the actin gene by a 14-aa linker (ASSGGSGSGGSGGA) to allow the thymosin-β4 enough flexibility to occupy the barbed end binding site on the actin monomer, thus preventing the actin from polymerizing with itself or native S*f*9 cell actin. A HIS6 tag was added at the C terminus for purification on a nickel affinity column. The final native residue of actin is Phe, which provides a site for chymotrypsin cleavage to remove the linker, thymosin-β4, and the HIS6 tag completely. PfAct1 monomers were purified on a gel filtration column (Superdex 200 10/300 GL, GE Lifesciences 28990944) equilibrated in 5 mM Tris pH 8.0 at 4 °C, 0.2 M NH_4_Ac, 0.2 mM CaCl_2_, 0.2 mM NaATP, 0.5 mM DTT. The peak monomeric fractions were converted to actin-MgATP by incubation with a 2-fold molar excess of MgCl_2_ and 200 μM EGTA for 10 min at 4 °C. A 1.1-fold molar excess of jasplakinolide (Thermo Fisher J7473) was added to the PfAct1-MgATP, followed by addition of one-tenth volume of a 10× polymerization buffer (100 mM imidazole, pH 7.4, 500 mM KCl, 40 mM MgCl_2_, 10 mM EGTA, 10 mM DTT), and incubation at 37°C for 1 h. Excess ATP was removed by dialysis versus 10 mM imidazole, pH 7.4, 50 mM KCl, 4 mM MgCl_2_, 1 mM EGTA, 1 mM DTT.

### Optimized sample preparation and image acquisition for cryo-EM

Various PfMyoA constructs were screened in conjunction with Skeletal F-actin, *P. falciparum* actin 1 (PfAct1) stabilized or not by JAS. These constructs included the full-length protein and the motor domain. In our hands, the best samples were generated when myosin was incubated in solution with the F-actin sample and vitrified within 15 min. Thus, four grids were generated at a time. In general, we applied 5 μl of filamentous actin/myosin solution to plasma cleaned holey carbon grid (C-flats 2/2, Protochips), incubated it for 1 min and manually blotted with filter paper and achieved vitrification by plunging into LN_2_ cooled, liquified ethane using an in-house designed cryo-plunger.

Screening for the best sample mixture ratios and blotting conditions was performed on a T12 Tecnai Spirit electron microscope (ThermoFisher Scientific) equipped with a 4Kx4K Eagle camera (ThermoFisher Scientific), operated at a voltage of 120 kV. Data sets were acquired on a Titan Krios electron microscope (ThermoFisher Scientific) equipped with an extra-high brightness field emission gun (X-FEG) and operated at a voltage of 300 kV. Although the sample preparation protocol was optimized, we had to screen for usable grids and grid squares manually. Images were recorded with a back-thinned 4 K×4 K Falcon 2 (ThermoFisher Scientific) direct detection camera under minimal dose conditions using the automatic data collection software EPU (ThermoFisher Scientific). Within each selected grid hole, two positions were imaged, each with a total exposure time of 1 s. Seven frames with a total electron dose of ~60 electrons per Å^2^ were collected. A total of 6073 image stacks were collected in five separate, independent imaging sessions. The nominal magnification of 75,000 corresponds to a pixel size of 1.035 Å. The defocus range of the data sets was 0.7–2.8 μm.

### Image processing and reconstruction

Dose weighting and motion correction were applied using MotionCor2^42^ with anisotropic motion correction using 5 × 5 patches. The initial defocus was estimated either using Gctf^43^ or CTFFIND4^44^, depending on the imaging session. The data was then processed with the helical reconstruction routines in RELION3^25,45^. Briefly, the helices were divided into overlapping boxes that were essentially treated as individual, independent particles (with modified Bayesian prior accounting for constraints implied by helicity) to allow sorting of the segments into different conformations and selecting the most well-defined of the conformations present in the sample, a prerequisite for reaching high resolution. A total of 509,035 filament segments were extracted using a box size of 336 × 336 pixels from 41,940 manually picked filaments. Two-dimensional reference-free classification for the data set was carried out in RELION3 to eliminate bad segments reducing the number of segments from 509,035 to 464,646. As an initial model, an in-house rabbit skeletal actin reconstruction filtered to 40-Å resolution was used. An initial reconstruction at 5.1 Å showing clear and detailed density for PfMyoA was obtained. After several rounds of 3D classification and refinement followed by manual removal of bad particles and careful enrichment of segments showing clear decoration using the initial reconstruction as a reference, the estimated resolution of the reconstruction reached 3.8 Å after processing using the 0.143 FSC cutoff gold-standard procedure implemented in RELION3. The processing included RELION3-based CTF refinement, B-factor sharpening, and application of a soft-edged mask generated in RELION3 corrected for helical edge effects using pyCoAn, an extended python version of CoAn^46^. Additional sharpening was applied using the auto-sharpening routine in Phenix^47,48^. The reconstruction was then symmetrized within pyCoAn using the helical parameters refined by RELION3. Local resolution estimates were calculated with ResMap^49^.

### Model building and refinement

The atomic models of the PfAct1 filament stabilized by JAS (PDB code 5OGW; https://www.rcsb.org/structure/5OGW) and PfMyoA in the Rigor-like state (PDB code 6I7D chain D; https://www.rcsb.org/structure/6I7D) as starting points. The models were independently fitted into the map with the function “fit in map” in UCSF Chimera^50^. Building of Loop2 and manual refinement of the model was performed with Coot^51^. A first step of real space refinement was performed with Phenix^48^. In order to improve the statistics at the interface and to compute realistic interactions, we performed an independent run of molecular dynamics. The system was built with the CHARMM-GUI^52,53^, using the Quick MD Simulator module. Only the actin binding elements of PfMyoA and surfaces of the PfAct1 filament interacting with PfMyoA were relaxed in a box containing explicit water (TIP3P) and salt (150 mM KCl) in the CHARMM36 force field, the rest of the model being fixed during all the duration of the simulation. The duration of the simulation was 10 ns in GROMACS (version 5.1.4)^54^. The output of the simulation was then submitted to another run of real space refinement. A final run of reciprocal space refinement was performed with Refmac^55^. To improve the overall consistency between the models used for comparison, the published models of the Myo6 and Myo1b Rigor states were subjected to reciprocal space refinement with Refmac. Quality indicators, including MolProbity^56^ and EMRinger^57^ scores, were calculated with Phenix. The additional refinement step improved the EMRinger score of Myo6 from 0.11 to 0.36 and the EMRinger score of Myo1b from 1.41 to 1.68. The Rigor state of NM2c was already refined with Refmac so the additional step was not necessary for that model.

### Interface analysis

All the actomyosin interfaces were analyzed automatically with the software PDBsum^58^ for protein-protein interfaces. The automatic analysis was complemented by a manual analysis to check long-range interactions with a cut-off of 4 Å. Areas of interaction were calculated with PISA^59^.

### Motility assays

For ensemble motility assays with PfMyoA and phosphorylated smooth muscle myosin, MgATP insensitive heads were removed by a 20 min spin at 350,000 *g* in the presence of 1.5 mM MgATP and a three-fold molar excess of actin. Myosin concentrations were determined using the Bio-Rad Protein assay. For PfMyoA motility assays, the following solutions were added to a nitrocellulose-coated flow cell in 15 µl volumes. 0.5 mg/ml biotinylated bovine serum albumin (BSA) in buffer A (100 mM KCl, 25 mM imidazole pH 7.5, 1 mM EGTA, 4 mM MgCl_2_, and 10 mM DTT) was added and incubated for 1 min. Three additions of 5.0 mg ml^−1^ BSA in buffer A were followed by a 2 min wait. 25 µg ml^−1^ neutravidin (Thermo Fischer Scientific) in buffer A was added for 1 min and then rinsed three times with buffer A. 120 µg/ml PfMyoA with a C-terminal biotin tag^24^ was flowed into the cell and incubated for 1 min followed by 3 rinses with buffer A. Skeletal, smooth^60^ and JAS stabilized PfAct1 filaments were incubated for 30 s followed by one rinse with buffer A and one rinse with buffer B (buffer A plus chromobody, 0.5% (w/v) methylcellulose, 25 µg ml^−1^ PfELC and 25 µg ml^−1^ PfMTIP and oxygen scavengers (50 µg ml^−1^ catalase (Sigma), 125 µg ml^−1^ glucose oxidase (Sigma), and 3 mg ml^−1^ glucose). 50 nM chromobody was used to visualize skeletal and smooth actins; 20 nM chromobody was used for PfAct1 filaments. Motility was initiated by adding buffer B containing 2 mM MgATP twice to the flow cell, followed by a 1 min wait for temperature to equilibrate to 30 °C on the microscope. Unstabilized PfAct1 filaments (freshly prepared for each experiment by polymerizing at 8 µM for 10 min at 37 °C), to which 20 nM actin chromobody and 2 mM MgATP were added, was directly loaded into the flow cell with no subsequent washes.

Full-length phosphorylated smooth muscle myosin^60^ was directly adhered to the nitrocellulose-coated coverslip. 15 µl of 100 µg ml^−1^ myosin in 300 mM KCl buffer C (25 mM imidazole pH 7.5, 1 mM EGTA, 4 mM MgCl_2_, and 10 mM DTT) was flowed into the cell and incubated for 1 min. Three 15 µl additions of 5.0 mg ml^−1^ BSA in 300 mM KCl buffer C were followed by a 2 min wait, and then three 15 µl rinses with 25 mM KCl buffer C. Skeletal, smooth and JAS stabilized PfAct1 filaments were incubated for 30 s followed by three 15 µl rinses with 90 mM KCl buffer C. Next, one rinse of buffer D (90 mM buffer C plus chromobody, 0.7% (w/v) methylcellulose, oxygen scavengers (50 µg ml^−1^ catalase (Sigma), 125 µg ml^−1^ glucose oxidase (Sigma), and 3 mg ml^−1^ glucose). 50 nM chromobody was used to visualize skeletal and smooth actins; 20 nM chromobody was used for PfAct1 filaments. Motility was initiated by adding buffer D containing 1 mM MgATP twice to the flow cell and waiting 1 min for temperature to equilibrate to 30 °C on the microscope. Unstabilized PfAct1 filaments (freshly prepared for each experiment by polymerizing at 8 µM for 10 min at 37 °C), to which 20 nM actin chromobody and 1 mM MgATP were added, was directly loaded into the flow cell with no subsequent washes. Filaments were automatically tracked and analyzed with the Fast Automated Spud Trekker analysis program (FAST, Spudich laboratory, Stanford University, http://spudlab.stanford.edu/fast-for-automatic-motility-measurements). Reported speeds are from a fit to a Gaussian curve. To assess the significance of differences in speed, a two-tailed *z*-test was chosen because it is ideal for determining whether two population means are different when the variances are similar, and the sample size is large. Samples were assumed to be independent of one another and the populations from which the samples were taken were assumed to be normally distributed. Actin filaments were visualized using a Nikon ECLIPSE Ti microscope.

### Reporting summary

Further information on research design is available in the Nature Research Reporting Summary linked to this article.

## Supplementary information

Supplementary Information

Reporting Summary

## Data Availability

Data supporting the findings of this manuscript are available from the corresponding authors upon reasonable request. A reporting summary for this Article is available as a Supplementary Information file. The atomic models and cryo-EM maps are available in the PDB^61^ and EMDB databases^62^, under accession numbers 7ALN and EMD-11818 respectively.
