# Supplementary material for: The actomyosin interface contains an evolutionary conserved core and an ancillary interface involved in specificity
Source: Nat Commun. 2021 Mar 25;12:1892. doi: 10.1038/s41467-021-22093-4 (PMC7994445; doi:10.1038/s41467-021-22093-4)
Supplement: Supplementary file 1 — Supplementary Information [file 41467_2021_22093_MOESM1_ESM.pdf]

Supplementary Information for:

The Actomyosin interface contains an evolutionary conserved core and an ancillary interface involved in specificity

Julien Robert-Paganin[1], Xiao-Ping Xu[2], Mark F. Swift[2], Daniel Auguin[1,3], James P. Robblee[4], Hailong Lu[4], Patricia M. Fagnant[4], Elena B. Krementsova[4], Kathleen M. Trybus[4], Anne Houdusse[1]*, Niels Volkmann[2,5]*, and Dorit Hanein[1,6]

[1] Structural Motility, Institut Curie, CNRS, UMR 144, F-75005 Paris, France
[2] Scintillon Institute, San Diego, CA USA 92121
[3] Laboratoire de Biologie des Ligneux et des Grandes Cultures (LBLGC), Université d'Orléans, INRAE, USC1328, 45067 Orléans, France
[4] Department of Molecular Physiology & Biophysics, University of Vermont, Burlington, VT USA 05446
[5] Structural Image Analysis Unit, Department of Structural Biology & Chemistry, Institut Pasteur, CNRS UMR 3528, 75015 Paris, France
[6] Structural Studies of Macromolecular Machines in Cellulo Unit, Department of Structural Biology & Chemistry, Institut Pasteur, CNRS UMR 3528, 75015 Paris, France

* Co-corresponding authors Niels Volkmann and Anne Houdusse:
Emails: niels.volkmann@pasteur.fr; anne.houdusse@curie.fr

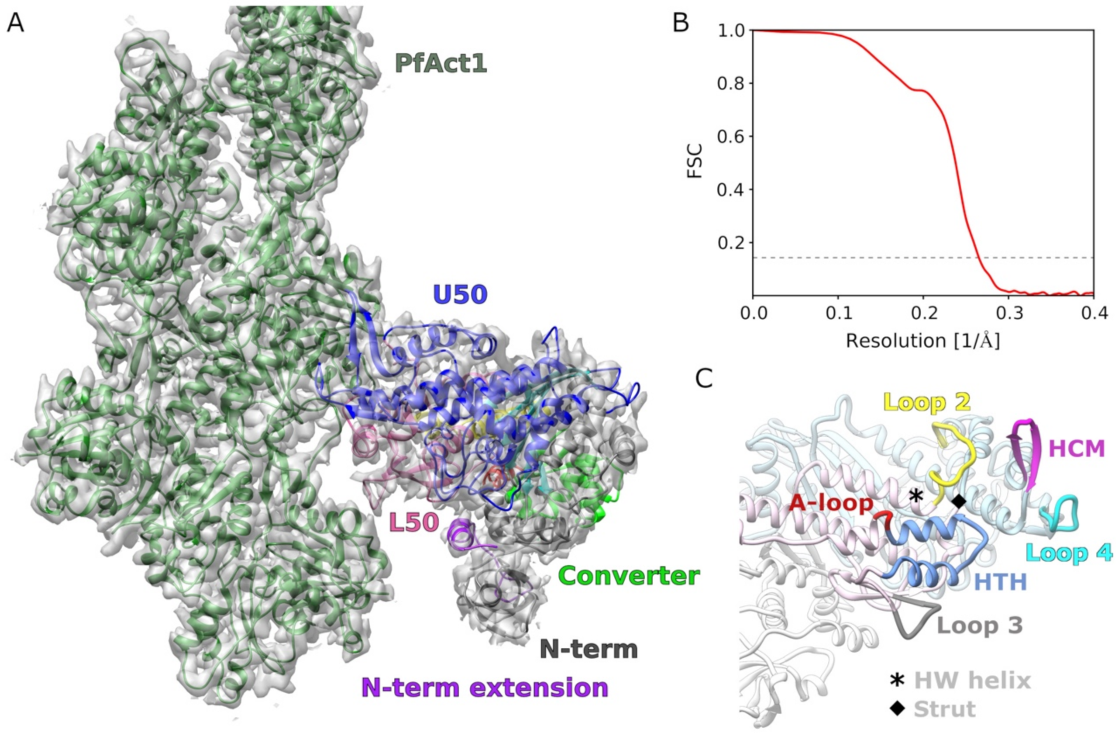

Supplementary Figure 1: *Structure of the PfMyoA motor domain/PfAct1 filament assembly* **(A)** Overview of the PfAc1/PfMyoA atomic model inside the density map with structural elements of PfMyoA colored in. The pointed end of the filament is up. 'L50' denotes 'lower 50kDa domain'. 'U50' denotes 'upper 50kDa domain'. **(B)** Fourier shell correlation (FSC) curve between two independently derived reconstruction using random half data sets. The 0.143 cutoff (dashed line) indicates a resolution of 3.8 Å. **(C)** Atomic model of PfAct1-bound PfMyoA motor domain with the primary structural elements participating in interactions colored distinctly and labeled (see also supplemental Table 2). 'HCM' denotes hypertrophic cardiomyopathy loop', 'HTH' denotes 'helix-turn-helix motif', and 'A-loop' denotes 'Activation loop'. The element that bridges the cleft between U50 and L50 ('Strut') is labeled with a diamond. The helix that follows Loop 2 in L50 ('HW helix') is labeled with an asterisk. This orientation is turned 180º around the filament axis from the orientation in A.

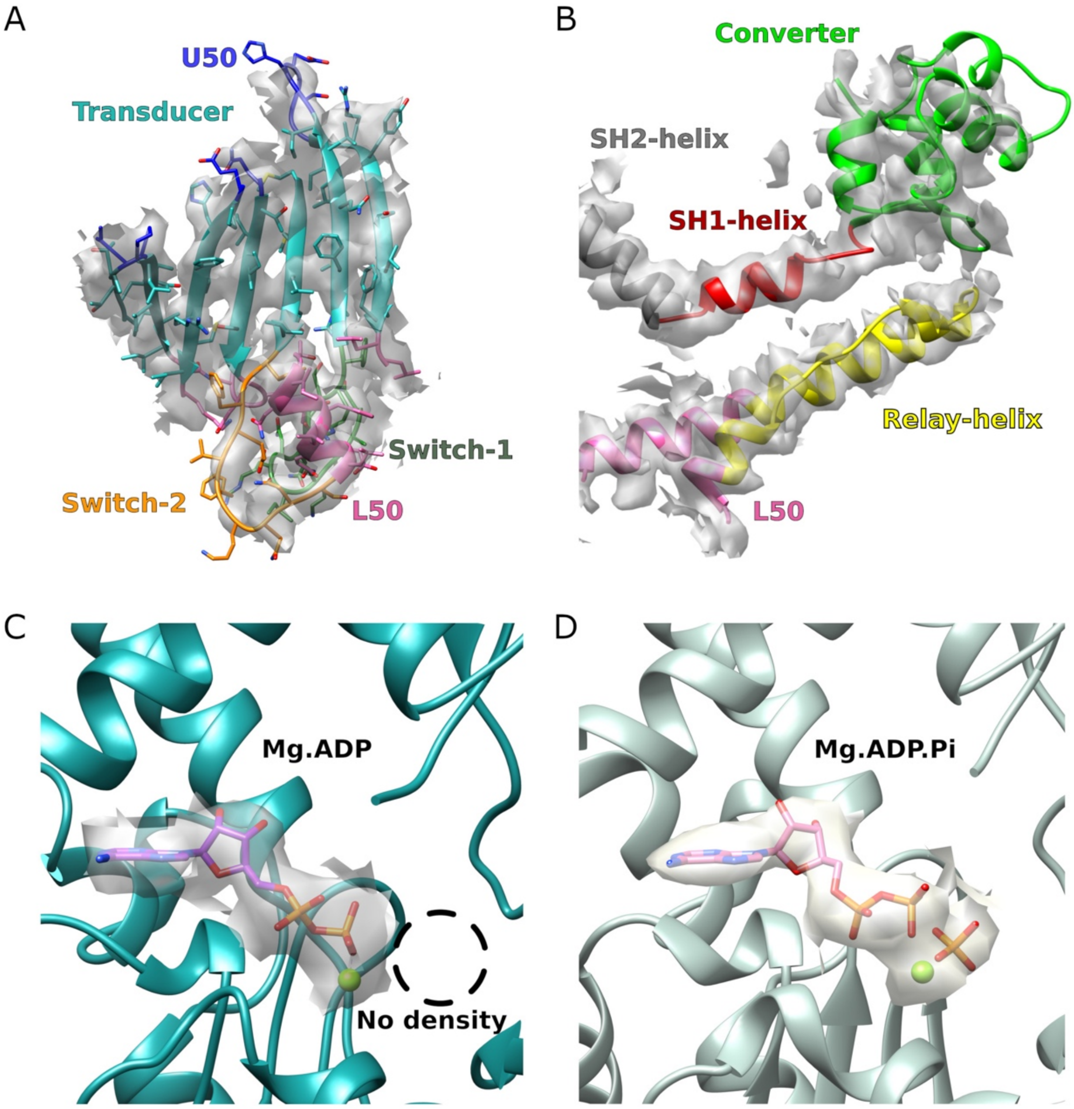

Supplementary Figure 2: *Structural details of the PfMyoA-PfAct1 assembly* **(A, B)** Density map and atomic model of the connectors within the motor domain of PfMyoA. The region close to the Transducer and the active site is shown in A. The region close to the Converter, the SH1-helix, and the Relay is shown in B. **(C)** MgADP is present in PfAct1 monomers from the PfActomyoA structure. No density is present at the place of the Pi. **(D)** In order to compare, the density of the nucleotide from an actin filament with bound MgADP.Pi (PDB code 6FHL), where the Pi is undeniably visualized in the density map.

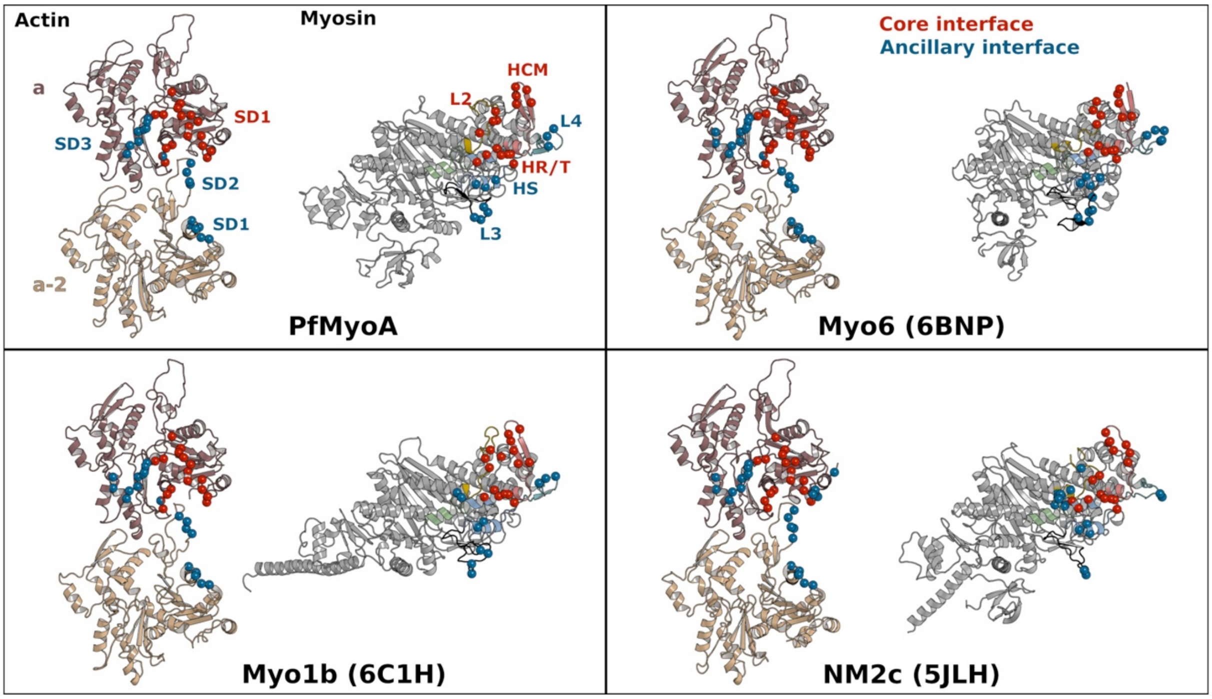

Supplementary Figure 3: *Actomyosin footprint analysis details.* Open book representation of the different structures of Rigor actomyosin complexes solved at high resolution: PfMyoA, myosin 6 (Myo6), myosin 1b (Myo1b), non-muscle myosin 2c (NM2c). Both on actin and on the myosin, residues part of core interface (in red) and ancillary interface (in blue) are shown as spheres. The hypertrophic cardiomyopathy loop (HCM), loop 2 (L2), loop 3 (L3) and loop 4 (L4) and the helix-turn-helix motif of myosin are labeled in the PfMyoA panel, colored according to whether the respective structural element primarily contributes to the core or the ancillary interface. The helix-turn-helix motif is split into the HR helix and the turn (HR/T) and the HS helix (HS). The subdomains of actin (SD) are also labeled and colored according to their primary contribution.

Supplementary Table 1. *Statistics for data acquisition, processing, and model refinement*

**Data collection**	
Microscope	Titan Krios
Voltage [kV]	300
Detector	Falcon II
Magnification	75,000
Exposure parameters	
Total dose [$e^-/Å^2$]	60
Exposure time [s]	1
Pixel size [Å]	1.035
Defocus range [µm]	-0.7 to -2.8

**Data processing**	
Images used	6,073
Initial segments	509,035
Final segments	464,646
Helical symmetry	
Rise [Å]	27.3
Twist [º]	-168.1
Resolution [Å]	3.8
FSC threshold	0.143
Sharpening B-factor [$Å^2$]	-93

**Refinement**	
Initial models [PDB IDs]	5ogw, 6i7d (D)
Non-hydrogen atoms	20,498
Model resolution [Å]	3.8
FSC threshold	0.5
RMSD deviations	
Bond lengths [Å]	0.008
Bond angles [º]	0.910
Rotamer outliers [%]	0.22
Mean B-factor [$Å^2$]	82.2

**Validation**	
Molprobity score	1.74
Clash score	8.84
Ramachandran plot	
Favored [%]	95.84
Allowed [%]	4.12
Disallowed [%]	0.04
CC (mask)	0.82
CC (volume)	0.80
EMringer score	2.71

Supplementary Table 2. *Footprint analysis of Rigor actomyosin interactions*

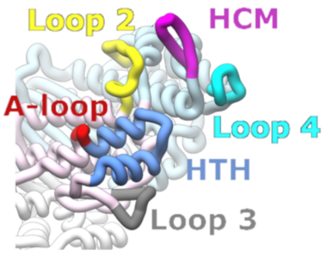

Table S2A: Core Interface (most conserved among Rigor cryo-EM structures)					
HCM loop	*Actin residues*	*PfMyoA (K410-R422)*	*Myo6 (R393-V409)*	*NM2c (P418-A430)*	*Myo1b (R327-T339)*
SD3.A	^{Pf}D26/^{Sk}D27; ^{Pf}A27/^{Sk}A28<br>^{Pf}P28/^{Sk}P29; ^{Pf}V31/^{Sk}V32	T412; V413<br>A414; G415	M395<br>L396	I420; K421<br>V422; G423	V329; E330<br>A331
	^{Pf}E335/^{Sk}E336	T412; I419	R393; M395<br>V409	I420; V427<br>Q428; K429	R327; V329<br>T338
	^{Pf}Y338/^{Sk}Y339	A414; G415<br>***D417 (mut)***	T397	V422; V427	A331; E334
HR helix [HTH]	*Actin residues*	*PfMyoA (S537-C546)*	*Myo6 (G524-N533)*	*NM2c (G549-C558)*	*Myo1b (G455-C464)*
SD1.A	^{Pf}S351/^{Sk}S352; ^{Pf}T352/^{Sk}T353	Y541		A552 ;L553	
	^{Pf}L350/^{Sk}L351; ^{Pf}S351/^{Sk}S352<br>^{Pf}T352/^{Sk}T353	D544	E531	E556	E462
HTH turn [HTH]	*Actin residues*	*PfMyoA (L547-T552)*	*Myo6 (R534-S539)*	*NM2c (W559-T564)*	*Myo1b (L465-T471)*
SD2.A-2	^{Pf}G47/^{Sk}G48	A548		F560	R466
SD1.A	^{Pf}I346/^{Sk}I347; ^{Pf}L350/^{Sk}L351	L547	R534	W559	
	^{Pf}S349/^{Sk}S350	L547	R534	W559	L465
	^{Pf}L350/^{Sk}L351	A548	L535	F560	
SD3.A	^{Pf}Y144/^{Sk}Y145; ^{Pf}G147/^{Sk}G148<br>^{Pf}R148/^{Sk}R147; ^{Pf}T149/^{Sk}T150	P549<br>G550	P536<br>Q537	P561<br>K562	P467<br>G468
Loop 2	*Actin residues*	*PfMyoA (L624-I641)*	*Myo6 (F621-V643)*	*NM2c (I637-V670)*	*Myo1b (L553-A570)*
SD1.A	^{Pf}G24/^{Sk}G25; ^{Pf}D25/^{Sk}D26<br>^{Pf}D26/^{Sk}D27	G633<br>K634	A635; G636<br>K637; L638	R663; M666<br>F667	L564; K565<br>R566
	^{Pf}S345/^{Sk}S346; ^{Pf}I346/^{Sk}I347	K634		F667	R566
	^{Pf}S349/^{Sk}S350	K637		F667	R566
Strut	*Actin residues*	*PfMyoA (K602-G607)*	*Myo6 (N598-M603)*	*NM2c (M615-D620)*	*Myo1b (N531-R536)*
SD1.A	^{Pf}D26/^{Sk}D27	R606			R536

**Table S2B: Ancillary interface (not conserved among Rigor cryoEM structures)**					
**HS helix [HTH]**	*Actin residues*	*PfMyoA (D553-K565)*	*Myo6 (D540-H551)*	*NM2c (D565-G577)*	*Myo1b (D472-A484)*
SD2.A-2	^{Pf}G47/^{Sk}G48; ^{Pf}M48/^{Sk}M49 **^{Pf}E49/^{Sk}G50; ^{Pf}E50/^{Sk}Q51**	K555; S558 T562	H542; S545 A546; Q549	K571 Q574	K478 Q481
	^{Pf}K51/^{Sk}K52			E570	
**Loop 4**	*Actin residues*	*PfMyoA (E369-A380)*	*Myo6 (E354-N363)*	*NM2c (R382-T390)*	*Myo1b (P288-K299)*
SD3.A	^{Pf}R148/^{Sk}R149	S376			L295
	**^{Pf}E312/^{Sk}D313; ^{Pf}T315/^{Sk}Q316**		T358;G356	R384 (tropo)	N293
	^{Pf}K329/^{Sk}K330; ^{Pf}V330/^{Sk}I331 **^{Pf}V331/^{Sk}I332;** ^{Pf}A332/^{Sk}A333 ^{Pf}P333/^{Sk}P334; ^{Pf}P334/^{Sk}P335	G374; L375 S376	S357; T358 S359; G360	N385	N293; G294 L295
**A-loop**	*Actin residues*	*PfMyoA (E533-K536)*	*Myo6 (A520-V523)*	*NM2c (R543-P548)*	*Myo1b (N451-N454)*
SD1.A	^{Pf}S351/^{Sk}S352; ^{Pf}Q354/^{Sk}Q355 ^{Pf}Q355/^{Sk}Q356			P544 ; A545 N546 ; P548	N452 T453
	^{Pf}E4/^{Sk}E5			E542	
**Loop 3**	*Actin residues*	*PfMyoA (P572-F581)*	*Myo6 (I559-F577)*	*NM2c (R584-F594)*	*Myo1b (S491-F510)*
SD1.A-2	^{Pf}Y92/^{Sk}Y93; ^{Pf}N93/^{Sk}N94 ^{Pf}H88/^{Sk}H89	K574; V575 A576; S577	R561	H587	M493; L500 N501
	^{Pf}R96/^{Sk}R97	A576 V575	N570; I571 R572; E575	R589	H507
	^{Pf}R96/^{Sk}R97	V575	R561	L588	
	**^{Pf}A97/^{Sk}V98**; ^{Pf}A98/^{Sk}A99		R561	L588	N501
**Loop 2**	*Actin residues*	*PfMyoA (L624-I641)*	*Myo6 (F621-V643)*	*NM2c (I637-V670)*	*Myo1b (L553-A570)*
SD1.A	^{Pf}E4/^{Sk}E5			R663	
**HW helix**	*Actin residues*	*PfMyoA (G642-T659)*	*Myo6 (G644-T661)*	*NM2c (G671-T688)*	*Myo1b (G571-K588)*
SD1.A	^{Pf}E4/^{Sk}E5			Q672; K675	

Footprint analysis of Rigor actomyosin interactions **(A)** List of conserved interactions (core interface). **(B)** List of interactions that are not conserved among Rigor actomyosin complexes (ancillary interface). See also Fig. 4 and Fig. S3.

Myosin structural elements and actin subdomains are listed in the first column. The structural elements of myosin are colored according to supplementary figure 1C. The helix-turn-helix motif (HTH) is divided into the two helices (HR helix, HS helix) and the turn (HTH turn). 'A-loop' denotes 'activation loop'. Actin subdomains (SD) are indicated in bold italic and '.A' indicates the actin subunit with the main interface and '.A-2' the lower neighbor along the long-pitch helix if the pointed end is up (as in the figures). The second, grey column contains the actin residues partaking in interactions. The 'pf' and 'sk' superscripts indicate '*Plasmodium falciparum*' and 'skeletal' sequences respectively. Residues that show divergent replacements between the two are indicated in bold and red. Residues with conservative replacements are indicated in bold and blue. The remaining four columns list the myosin residues partaking in interactions with the actin residues in the corresponding row. The NM2c residue contacting tropomyosin (R384) is indicated in grey and labeled with '(tropo)'.

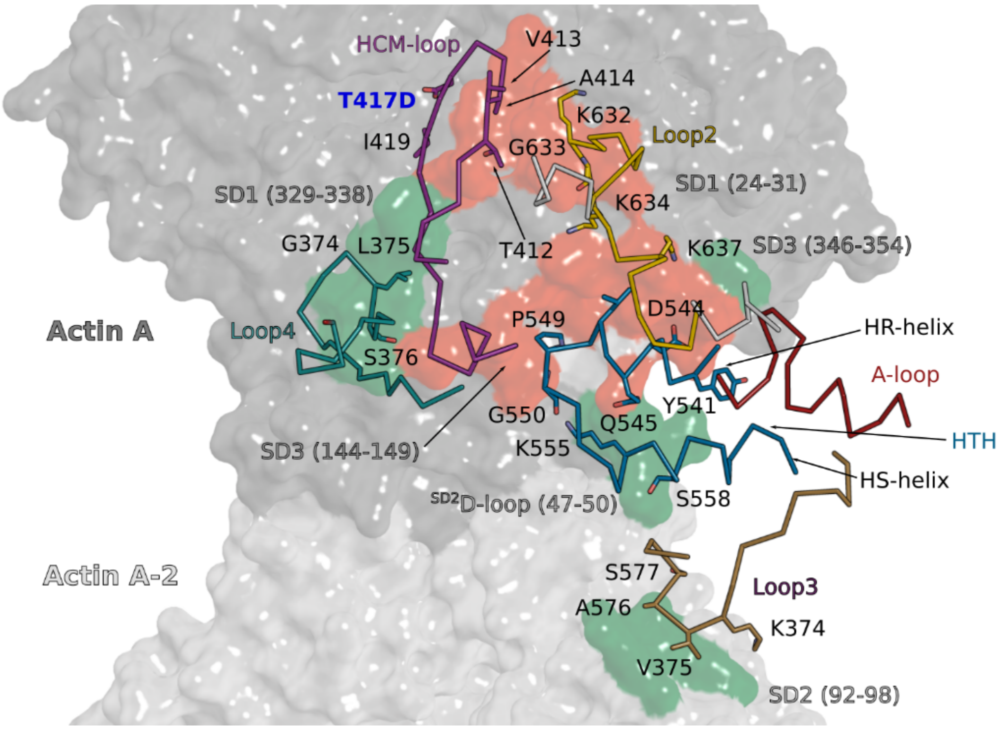

Supplementary Figure 4: *Pf-actomyosin interface details.* Two subunits of the PfAct1 filament model are shown in the back as surfaces. The core interface is colored in red and the ancillary interface is colored in green. Structural PfMyoA elements involved in interactions are shown as chain traces with selected residues marked and shown in full-atom stick-representation.

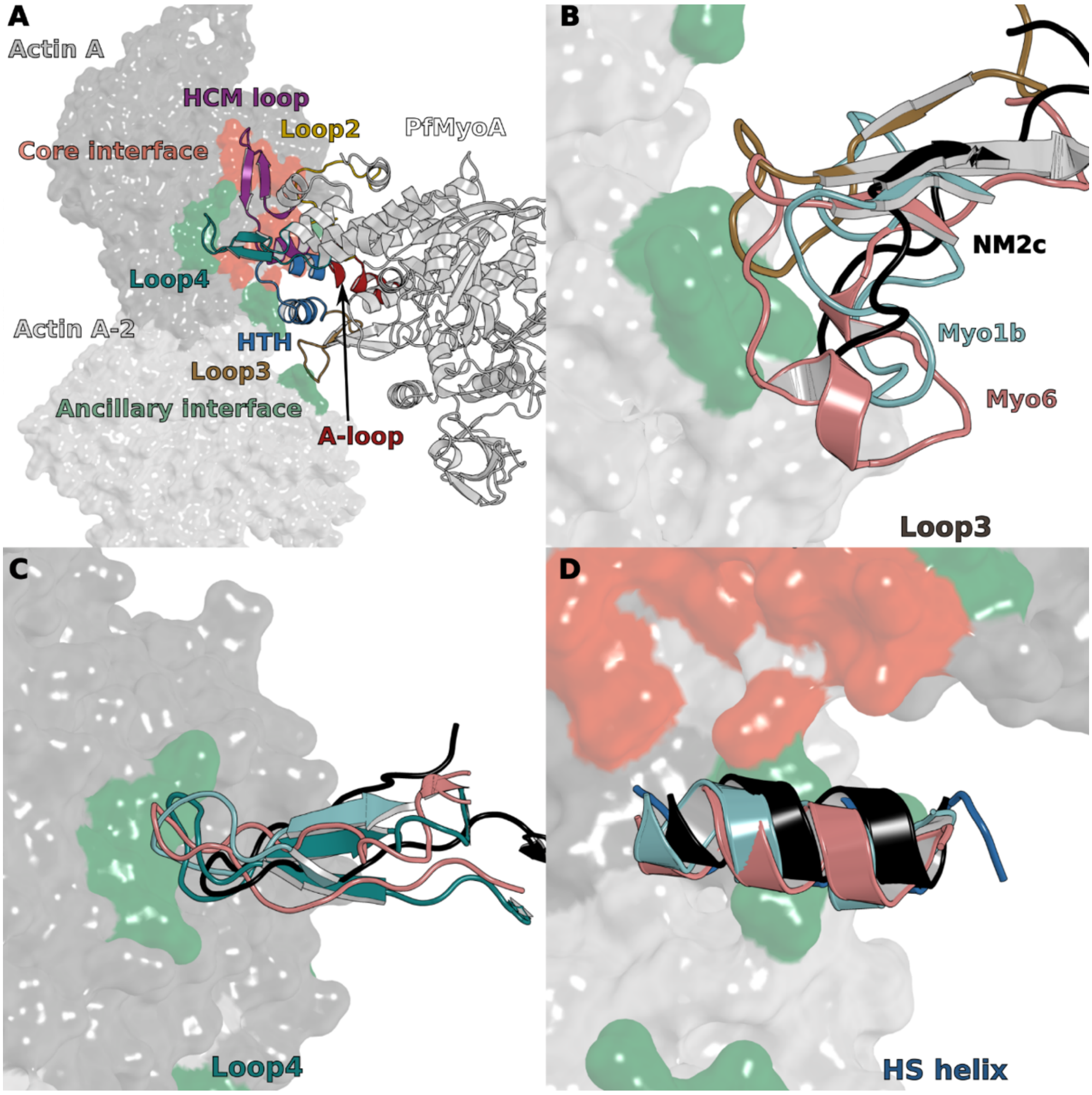

Supplementary Figure 5: *Variability at the Actomyosin interface* **(A)** overall view of the PfMyoA footprint. The Core interface is colored in red, the Ancillary interface is colored in green. **(B)**, **(C)** and **(D)** show the variability of the elements of the Ancillary interface in PfMyoA compared to NM2c (black), Myo6 (deep salmon) and Myo1b (light cyan). **(B)** shows Loop3, **(C)** shows Loop3 and **(D)** shows the HS helix of HTH.
