# Supplementary material for: The actomyosin interface contains an evolutionary conserved core and an ancillary interface involved in specificity
Source: Nat Commun. 2021 Mar 25;12:1892. doi: 10.1038/s41467-021-22093-4 (PMC7994445; doi:10.1038/s41467-021-22093-4)
Supplement: Supplementary file 2 — Reporting Summary [file 41467_2021_22093_MOESM2_ESM.pdf]

nature research

Corresponding author(s): Anne Houdusse and Niels Volkmann

Last updated by author(s): Feb 2, 2021

Reporting Summary

Nature Research wishes to improve the reproducibility of the work that we publish. This form provides structure for consistency and transparency in reporting. For further information on Nature Research policies, see our Editorial Policies and the Editorial Policy Checklist.

Statistics

For all statistical analyses, confirm that the following items are present in the figure legend, table legend, main text, or Methods section.

n/a	Confirmed	
☐	☒	The exact sample size (*n*) for each experimental group/condition, given as a discrete number and unit of measurement
☐	☒	A statement on whether measurements were taken from distinct samples or whether the same sample was measured repeatedly
☐	☒	The statistical test(s) used AND whether they are one- or two-sided<br>*Only common tests should be described solely by name; describe more complex techniques in the Methods section.*
☒	☐	A description of all covariates tested
☐	☒	A description of any assumptions or corrections, such as tests of normality and adjustment for multiple comparisons
☐	☒	A full description of the statistical parameters including central tendency (e.g. means) or other basic estimates (e.g. regression coefficient) AND variation (e.g. standard deviation) or associated estimates of uncertainty (e.g. confidence intervals)
☐	☒	For null hypothesis testing, the test statistic (e.g. *F*, *t*, *r*) with confidence intervals, effect sizes, degrees of freedom and *P* value noted<br>*Give P values as exact values whenever suitable.*
☒	☐	For Bayesian analysis, information on the choice of priors and Markov chain Monte Carlo settings
☒	☐	For hierarchical and complex designs, identification of the appropriate level for tests and full reporting of outcomes
☒	☐	Estimates of effect sizes (e.g. Cohen's *d*, Pearson's *r*), indicating how they were calculated

Our web collection on statistics for biologists contains articles on many of the points above.

Software and code

Policy information about availability of computer code

Data collection	EPU 1.20.1.1256 - single-particle data collection software (ThermoFisher Scientific)
Data analysis	MotionCor2 1.3.0 - motion correction; Gctf 1.06, CTFFIND4 4.1.5 - contrast transfer function estimation; RELION3 3.0.8 - helical/single-particle reconstrruction; pyCoAn 0.3.0 - mask application, helical averaging; Phenix 1.17.1 - sharpening, real-space refinment; Molprobity (via Phenix 1.17.1) - validation; EMRinger (via Phenix 1.17.1)- validation; Coot 0.8.9: modeling; UCSF Chimera 1.14 - fitting, visualization; CHARM-Gui 3.0 - simulation setup; GROMACS 5.1.4 - molecular dynamics simulation; Refmac 5.0.32 - reciprocal space refinement; PDBsum web version (http://www.ebi.ac.uk) - interface analysis; PISA 2.0.4 - interface area calculation; FAST 1.0.1 - motility assay analysis. RefMac 1.14 - local resolution.

For manuscripts utilizing custom algorithms or software that are central to the research but not yet described in published literature, software must be made available to editors and reviewers. We strongly encourage code deposition in a community repository (e.g. GitHub). See the Nature Research guidelines for submitting code & software for further information.

Data

Policy information about availability of data

All manuscripts must include a data availability statement. This statement should provide the following information, where applicable:

- Accession codes, unique identifiers, or web links for publicly available datasets
- A list of figures that have associated raw data
- A description of any restrictions on data availability

The atomic models and cryo-EM maps are available in the PDB and EMDB databases under accession numbers 7ALN (https://www.rcsb.org/structure/7ALN) and EMD-11818 (https://www.ebi.ac.uk/pdbe/entry/emdb/EMD-11818) respectively. Other publicly available data used in this study: PDB code 5OGW; https://www.rcsb.org/structure/5OGW; PDB code 6I7D chain D; https://www.rcsb.org/structure/6I7D.

Field-specific reporting

Please select the one below that is the best fit for your research. If you are not sure, read the appropriate sections before making your selection.

- [x] Life sciences
- [ ] Behavioural & social sciences
- [ ] Ecological, evolutionary & environmental sciences

For a reference copy of the document with all sections, see nature.com/documents/nr-reporting-summary-flat.pdf

Life sciences study design

All studies must disclose on these points even when the disclosure is negative.

Sample size	Motility assay: > 900 per motility experiment. The sample size in a motility experiment is the number of actin filaments observed during the course of the observation window, or movie. The filaments were counted by an analysis program used to track and measure filament movement (FAST, Spudich laboratory, Stanford University, http://spudlab.stanford.edu/fast-for-automatic-motility-measurements). This is an exact count of the number of observed and tracked filaments, so no statistical methods were used. The sample number, or filament count, was sufficiently large to generate two-sided, symmetrical Gaussian speed distributions.
Data exclusions	None excluded.
Replication	Two separate experiments per condition. All attempts at replication were successful and repeated, within the reported error, the published results.
Randomization	Randomization is not applicable to a motility assay because one 'sample' is prepared on a glass coverslip at which time nucleotide (ATP) is added to initiate actin filament movement. This 'sample' must be immediately observed to track and measure filament motion otherwise the nucleotide and proteins will degrade, thereby impacting filament movement. One 'sample' must be made and immediately observed/ measured. This process can be repeated, but not randomized.
Blinding	Blinding is not applicable to a motility assay because the filaments are tracked and measured by the FAST analysis software (FAST, Spudich laboratory, Stanford University, http://spudlab.stanford.edu/fast-for-automatic-motility-measurements), thereby eliminating any observer's bias that could occur when only selecting and tracking a specifically chosen subset of the total observed filaments.

Reporting for specific materials, systems and methods

We require information from authors about some types of materials, experimental systems and methods used in many studies. Here, indicate whether each material, system or method listed is relevant to your study. If you are not sure if a list item applies to your research, read the appropriate section before selecting a response.

Materials & experimental systems

n/a	Involved in the study
x	Antibodies
x	Eukaryotic cell lines
x	Palaeontology and archaeology
x	Animals and other organisms
x	Human research participants
x	Clinical data
x	Dual use research of concern

Methods

n/a	Involved in the study
x	ChIP-seq
x	Flow cytometry
x	MRI-based neuroimaging
